# Metabolomic profiling of overnight peritoneal dialysis effluents predicts the peritoneal equilibration test type

**DOI:** 10.1038/s41598-023-29741-3

**Published:** 2023-03-07

**Authors:** Hyo Jin Kim, Munki Choo, Hyuk Nam Kwon, Kyung Don Yoo, Yunmi Kim, Bodokhsuren Tsogbadrakh, Eunjeong Kang, Sunghyouk Park, Kook-Hwan Oh

**Affiliations:** 1 Department of Internal Medicine, Pusan National University Hospital, Pusan National University School of Medicine, Busan, Korea; 2 Biomedical Research Institute, Pusan National University Hospital, Busan, Korea; 3 Natural Product Research Institute, College of Pharmacy, Seoul National University, Seoul, Korea; 4 Department of Veterinary Biosciences, Faculty of Veterinary Medicine, University of Helsinki, Helsinki, Finland; 5 Department of Internal Medicine, Ulsan University Hospital, University of Ulsan College of Medicine, Ulsan, Korea; 6 Department of Internal Medicine, Inje University Busan Paik Hospital, Busan, Korea; 7 Laboratory Animal Team, Korea Institute of Radiological and Medical Sciences, Seoul, Korea; 8 Transplantation Center, Seoul National University Hospital, Seoul, Korea; 9 Department of Internal Medicine, Seoul National University Hospital, Seoul, Korea; 10 Department of Internal Medicine, Seoul National University College of Medicine, Seoul, Korea

**Keywords:** Peritoneal dialysis, Metabolomics

## Abstract

This study primarily aimed to evaluate whether peritoneal equilibration test (PET) results can be predicted through the metabolomic analysis of overnight peritoneal dialysis (PD) effluents. From a total of 125 patients, overnight PD effluents on the day of the first PET after PD initiation were analyzed. A modified 4.25% dextrose PET was performed, and the PET type was categorized according to the dialysate-to-plasma creatinine ratio at the 4-h dwell time during the PET as follows: high, high average, low average, or low transporter. Nuclear magnetic resonance (NMR)-based metabolomics was used to analyze the effluents and identify the metabolites. The predictive performances derived from the orthogonal projection to latent structure discriminant analysis (OPLS-DA) modeling of the NMR spectrum were estimated by calculating the area under the curve (AUC) using receiver operating characteristic curve analysis. The OPLS-DA score plot indicated significant metabolite differences between high and low PET types. The relative concentrations of alanine and creatinine were greater in the high transporter type than in the low transporter type. The relative concentrations of glucose and lactate were greater in the low transporter type than in the high transporter type. The AUC of a composite of four metabolites was 0.975 in distinguish between high and low PET types. Measured PET results correlated well with the total NMR metabolic profile of overnight PD effluents.

## Introduction

Peritoneal dialysis (PD) is a method of renal replacement therapy, and the peritoneal transport characteristics of patients with PD are evaluated to ensure appropriate prescription. The peritoneal equilibration test (PET) is a common method of examining peritoneal transport characteristics that measure the rate of solute transfer and water removal across the peritoneal membrane in patients with PD^1^. PET is usually performed between 4 and 8 weeks after initiation of PD^2,3^ and is repeated annually or when clinically necessary. The PET result is used to individualize the PD prescription by optimizing solute clearance and maximizing daily peritoneal ultrafiltration^4,5^, which is associated with the PD outcome^6,7^. Previous studies showed that a higher peritoneal transport status is associated with higher mortality and technique failure^8^. 2.5% dextrose PET (standard PET)^1^ or 4.25% dextrose PET (modified PET)^9^ can be used^10^.

Metabolomics is a field of “omics,” and is an important research field along with genomics, transcriptomics, and proteomics that reveal the function of genes or proteins. It studies metabolic processes, identifies important biomarkers related to metabolic features, and uncovers metabolic mechanisms^11,12^. Since human diseases or pathologic conditions are related to changes in metabolisms in the body, attempts to apply metabolomics to the discovery or identification of diagnostic biomarkers or therapeutic targets are increasing^13^. In particular, metabolomics has been widely applied to kidney-related diseases, since kidney-derived urine is easy to collect and reflects the status of the kidney^14–16^. Differences in metabolomic profiles of patients with pre-dialysis chronic kidney disease, hemodialysis, and PD have been evaluated^17^. There was a study showing differences in metabolomic profiles depending on the dialysis membrane in patients with hemodialysis^18^. A few studies have also been conducted with metabolomics for PD. In patients who developed encapsulating peritoneal sclerosis, distinct metabolomes were found in PD effluents compared to that in matched controls^19^. Changes in amino acids, amines, short-chain fatty acids, and sugars were observed prior to encapsulating peritoneal sclerosis development^19^. A previous study showed feasibility of metabolomic analysis of PD effluents from patients undergoing PET^20^, but the small number of patients necessitates further research. Although several recent metabolomics studies have been performed with mass spectrometry, probably because of the better availability and sensitivity of the platform, there are some considerations. Mass spectrometry requires solvent extraction, which may introduce disproportionate metabolite recovery and sample-to-sample variations^21,22^. Additionally, technical considerations, such as ion suppression and matrix effects, should be considered^23^. In comparison, nuclear magnetic resonance (NMR) is highly reproducible and quantitative. Biological liquid samples can be analyzed without solvent extraction and NMR is useful for analysis requiring sample recovery because the analysis is non-destructive^24,25^.

The PD effluent might be appropriate for metabolomic analysis because it is in direct contact with the site of action (peritoneal membrane) and reflects cell and tissue changes, possibly enabling the assessment of peritoneal transport characteristics in patients with PD. Thus far, there have been few studies on patients with PD using NMR-based metabolomics. With the fact that the PD effluent is in direct contact with the peritoneum, there might be significant differences in the metabolomic profile depending on the type of peritoneal membrane. The authors aimed to evaluate whether PET results can be predicted through the NMR-based metabolomic analysis of the overnight PD effluents and determine whether there are differences in metabolites depending on the PET types.

## Results

### Demographic characteristics of patients

**Figure 1:**
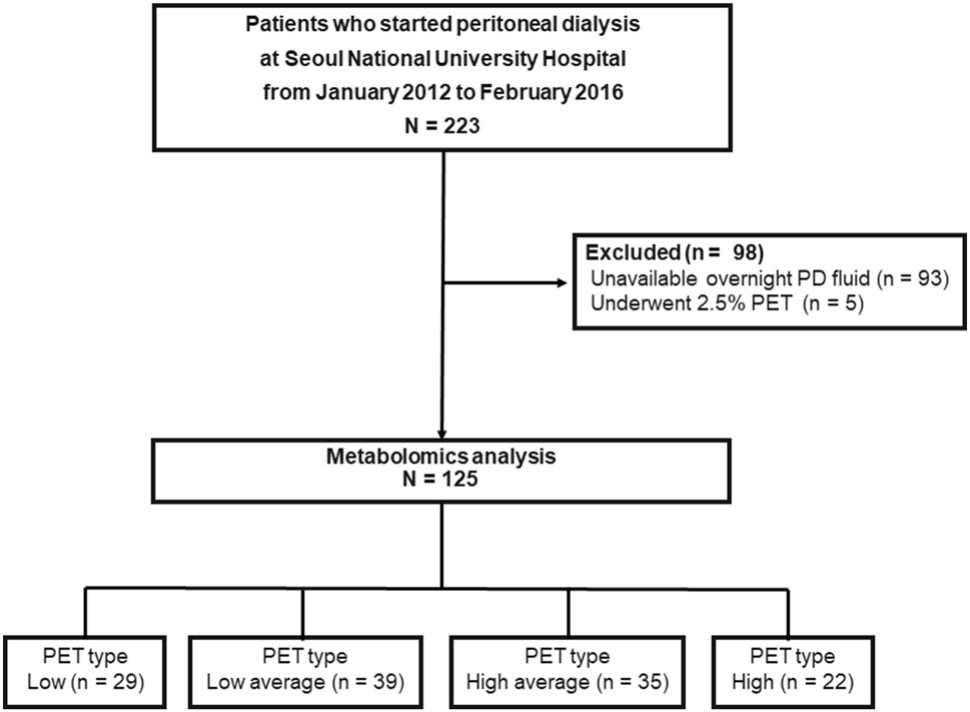
Patients in the study analyses. *PD* peritoneal dialysis, *PET* peritoneal equilibration test.

Of the 225 patients who started PD at Seoul National University Hospital from January 2012 to February 2016, 125 patients were finally analyzed (Fig. 1). Patients’ demographic characteristics according to PET types are presented in Table 1. Patients’ mean age was 44.2 ± 13.6 years, and 65 (52%) were men. Thirty-six (28.8%) and 94 (75.8%) patients had diabetes mellitus (DM) and hypertension (HTN), respectively. The high PET type was more common in patients with DM (*P* = 0.027). The serum albumin level was lower in the high PET type than in the other PET types (*P* = 0.044). The daily urine volume (*P* = 0.523) and Kt/V urea (*P* = 0.472) were similar among PET types.

**Table 1:** Demographic characteristics according to the PET types.

	Total (N = 125)	PET type				*P*-value
		Low (n = 29)	Low average (n = 40)	High average (n = 34)	High (n = 22)	
Age (mean ± SD)	44.2 ± 13.6	38.1 ± 12.4	46.7 ± 13.6	46.1 ± 13.9	44.7 ± 13.3	0.048
Sex, male, n (%)	65 (52.0)	14 (48.3)	22 (55.0)	17 (50.0)	12 (54.5)	0.937
BMI (kg/m^2^)	22.4 ± 2.8	22.3 ± 2.6	22.3 ± 2.7	22.8 ± 2.7	21.9 ± 3.2	0.679
DM, n (%)	36 (28.8)	6 (20.7)	11 (27.5)	7 (20.6)	12 (54.5)	0.027
HTN, n (%)	94 (75.8)	19 (67.9)	31 (77.5)	26 (76.5)	18 (81.8)	0.689
Primary renal disease, n (%)						0.187
DM	30 (24.0)	5 (17.2)	8 (20.0)	5 (14.7)	12 (54.5)	
HTN	23 (18.4)	6 (20.7)	6 (15.0)	8 (23.5)	3 (13.6)	
GN	48 (38.4)	11 (37.9)	16 (40.0)	15 (44.1)	6 (27.3)	
ADPKD	2 (1.6)	1 (3.4)	0 (0)	1 (2.9)	0 (0)	
Others	11 (8.7)	2 (6.9)	5 (12.5)	3 (8.8)	1 (4.5)	
Unknown	11 (8.8)	4 (13.8)	5 (12.5)	2 (5.9)	0 (0)	
4 h-D/P creatinine	0.71 ± 0.12	0.55 ± 0.05	0.68 ± 0.03	0.76 ± 0.03	0.89 ± 0.05	< 0.001
Urine volume (mL/day)	1047 ± 545	1051 ± 543	1092 ± 487	1094 ± 588	893 ± 591	0.523
Kt/V urea	2.2 ± 0.6	2.2 ± 0.5	2.2 ± 0.6	2.4 ± 0.6	2.2 ± 0.6	0.472
Hemoglobin (g/dL)	10.0 ± 1.8	9.5 ± 2.2	10.2 ± 1.5	10.2 ± 1.9	9.9 ± 1.4	0.376
Albumin (g/dL)	3.8 ± 0.6	3.9 ± 0.5	3.8 ± 0.5	3.8 ± 0.5	3.5 ± 0.6	0.044
Creatinine (mg/dL)	8.8 ± 3.9	9.4 ± 5.4	8.1 ± 2.4	9.2 ± 3.9	8.6 ± 3.8	0.553
CRP, median, (Q1, Q3) (mg/L)	0.20 (0.10, 0.50)	0.20 (0.10, 0.40)	0.20 (0.10, 0.50)	0.35 (0.10, 0.90)	0.20 (0.10, 1.40)	0.400

*PET* peritoneal equilibration test, *BMI* body mass index, *DM* diabetes mellitus, *HTN* hypertension, *GN* glomerulonephritis, *ADPKD* autosomal dominant polycystic kidney disease, *4 h-D/P creatinine* dialysate/plasma creatinine ratio at 4 h according to the peritoneal equilibration test, *CRP* C-reactive protein, *Q1* quartile 1, *Q3* quartile 3.

### Metabolomic profile and multivariate analysis of overnight PD effluents according to the PET types

**Figure 2:**
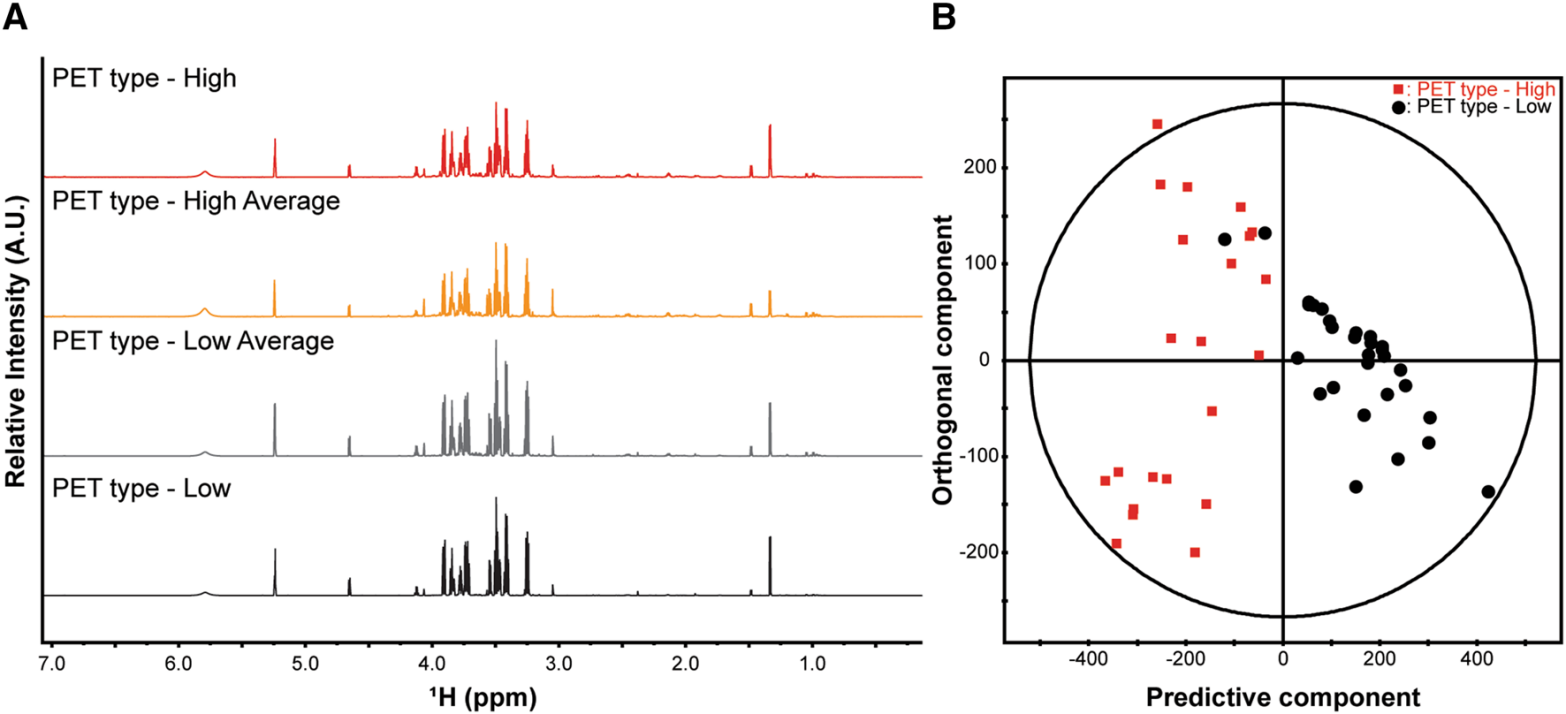
Representative NMR spectra obtained from overnight PD effluents and multivariate analysis between the PET types. (**A**) In total of 125 PD effluent samples were analyzed using 1D proton NMR spectra and representative samples from each group. A.U. stands for arbitrary unit, which is a relative unit of measurement used to show the intensity ratio in relation to a predetermined reference measurement. (**B**) OPLS-DA score plot derived from NMR spectra of overnight PD effluents between high and low PET types. OPLS-DA score plot showing the predictive component (X-axis) and the orthogonal component (Y-axis) of the model. The discrimination model was obtained with 1 predictive and 2 orthogonal components with the goodness of fit (R^2^) of 0.737 and the predictability (Q^2^) of 0.666. Each symbol represents the metabolic characteristic of a particular sample in the projected dimensions of predictive and 2 orthogonal components. The separation along the predictive component axis indicates the difference of interest (high versus low PET type) from the metabolic profile. *OPLS-DA* orthogonal projections to latent structure discriminant analysis, *NMR* nuclear magnetic resonance, *PET* peritoneal equilibration test.

NMR spectra were obtained to identify possible differences in metabolic profile among different PET types in 125 patients. Representative 1D proton NMR spectra are shown in Fig. 2A. Metabolites from the PD effluents were identified based on the chemical shift values and are shown in Supplemental Table 1. Multivariate analysis was performed to analyze the NMR data in a holistic manner. Unsupervised principal component analysis (PCA) was first conducted to evaluate the metabolic phenotypes to visualize the likenesses and differences across the complicated data sets (R^2^ = 0.926, Q^2^ = 0.809; Supplemental Fig. 1). The PCA analysis results confirmed differences among PET types. Then, supervised orthogonal projection to latent structure discriminant analysis (OPLS-DA) approach was performed to establish the difference between the groups and to find biomarkers in the presence of potentially confounding variables. As a result, only high and low PET types demonstrated meaningful differences, and there was no distinction in the other groups (Supplemental Table 2). The OPLS-DA model score plot indicated significant differences between the high and low PET types (R^2^ = 0.737, Q^2^ = 0.666; Fig. 2B).

### Discrimination of high and low PET types with metabolomic differences

**Figure 3:**
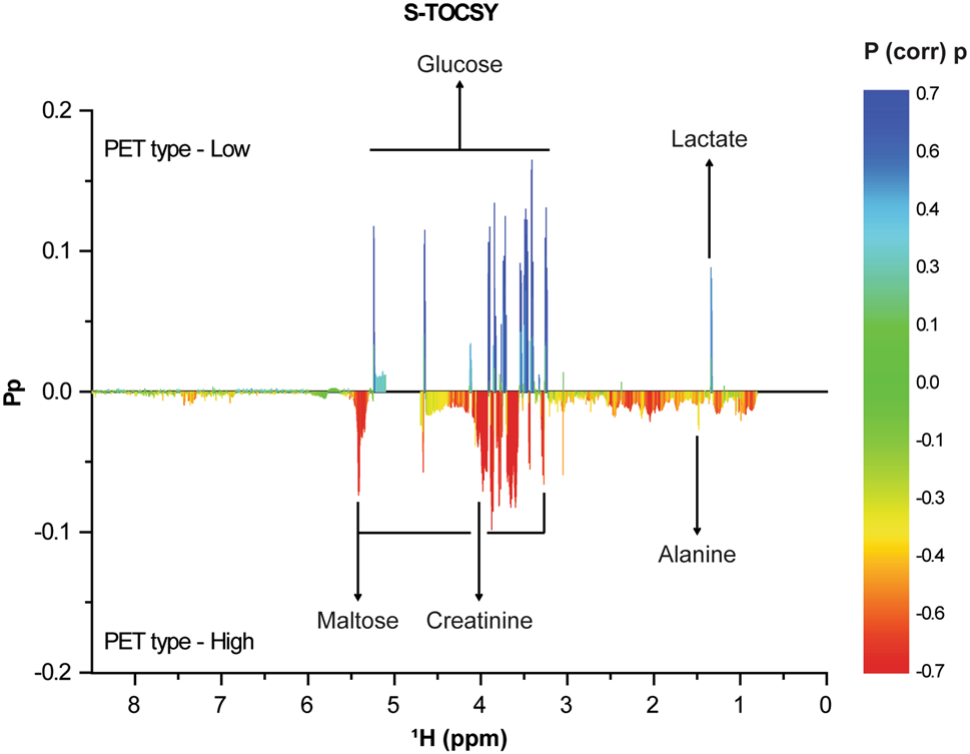
Identification of metabolites contributing to the discriminating model. S-TOCSY from the OPLS loading plot indicates the model coefficients for NMR variables. Signals are color-coded by the weights as a discriminator between the high and low PET types. Pp stands for modeled covariation, and P (corr) p stands for modeled correlation, as indicated by the color scale on the right. P (corr) p values for each of these signals indicate that multiple metabolite signals, rather than a single overwhelmingly prominent marker, contribute to differentiation. The metabolites that significantly distinguish the high and low PET types are annotated in the model coefficient plot. *S-TOCSY* statistical total correlation spectroscopy, *OPLS* orthogonal projections to latent structure, *NMR* nuclear magnetic resonance, *PET* peritoneal equilibration test.

**Figure 4:**
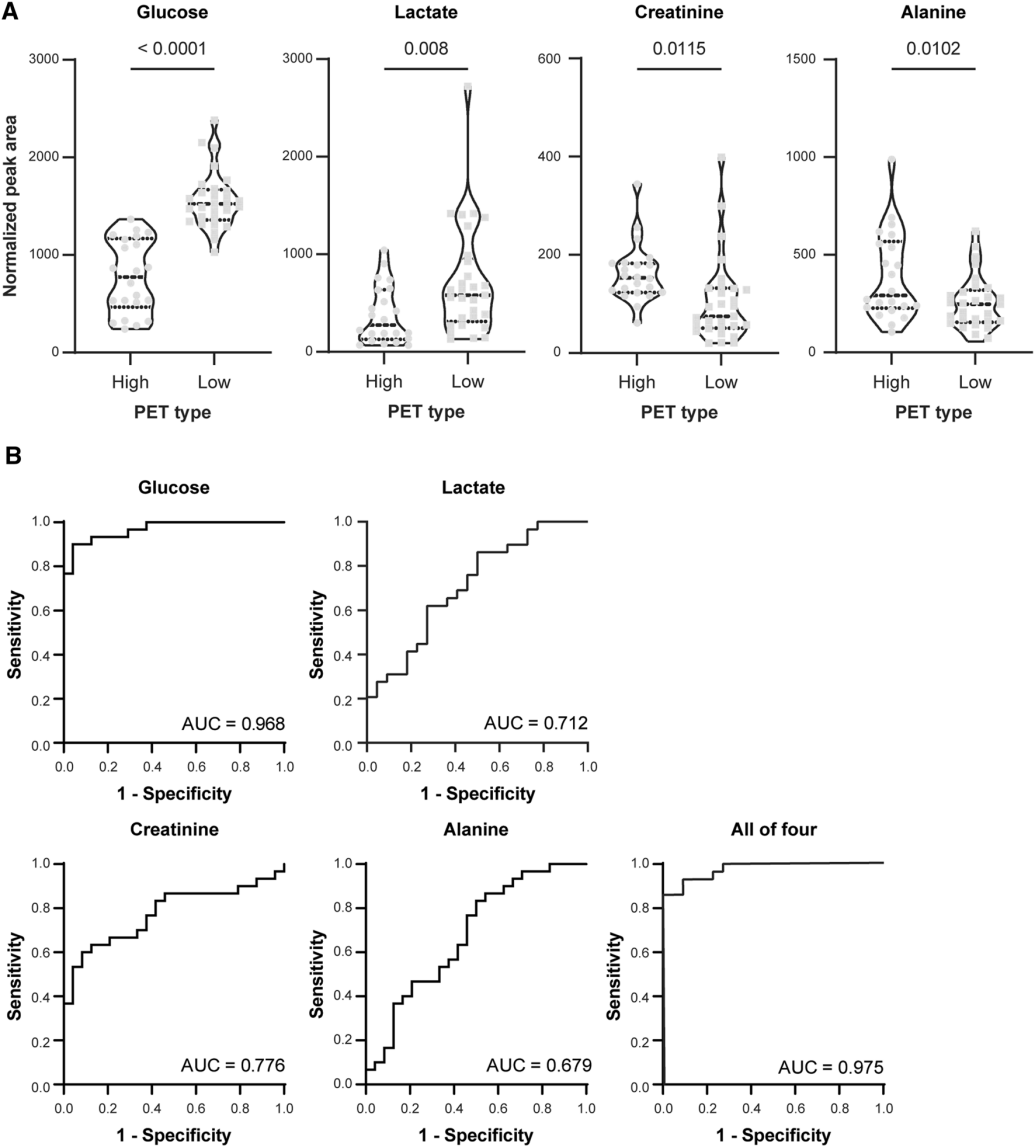
Statistical significance of the markers. (**A**) Levels of markers between the high and low PET types. Violin plots are presented with the median and quartiles. The levels were determined by the mean peak areas normalized by the total area from 1D proton NMR spectra in Fig. 2A. (**B**) PLS-DA-based ROC curves for the diagnosis of high and low PET types with the individual markers–glucose, lactate, creatinine, and alanine and the combination of the 4 markers. Statistical analysis of markers was performed using the Benjamini–Hochberg correction, and the resulting FDR* P*-values are indicated. *PET* peritoneal equilibration test, *NMR* nuclear magnetic resonance, *PLS-DA* partial least square-discriminant analysis, *ROC* receiver operating characteristic, *AUC* area under the curve, *FDR* false discovery rate.

Major contributing metabolites for the separation of PET types were identified by statistical total correlation spectroscopy (S-TOCSY; Fig. 3). Among those identified, we selected 5 metabolites, and they also had high Pp and P (corr) *P*-values identified (Fig. 3), which are more relevant to the differentiation, as candidate markers for distinguishing the high and low PET types. The relative concentrations of alanine (1.48 [doublet] ppm) and creatinine (4.07 [singlet] ppm) were greater in the high PET type than in the low PET type. Additionally, the relative concentrations of glucose (3.22 [multiplet], 3.38–3.91 [multiplet], 5.23 [doublet] ppm) and lactate (1.33 [doublet] ppm) were greater in the low PET type than in the high PET type depending on OPLS-DA loadings of the predictive latent variable. Moreover, maltose (3.26 [multiplet], 3.62–3.91 [multiplet], 4.64 [doublet], 5.4 [doublet, doublet] ppm) was high in the high PET type on S-TOCSY. This is because 11 patients with the high PET type received the icodextrin PD solution. Icodextrin from the peritoneal cavity to the circulation is promptly metabolized by amylase into small oligosaccharides, especially maltose^26^. A previous study that analyzed metabolites in patients with dialysis also confirmed the appearance of maltose in patients with PD using icodextrin dialysate^14^. If patients who used icodextrin were excluded, the maltose was not meaningful in the high PET type, so maltose was excluded. To confirm the validity of the markers found in this multivariate analysis, we also used the Benjamini–Hochberg correction. Alanine (1.48 ppm) and creatinine (4.07 ppm) levels were statistically higher in the high PET type, whereas glucose (3.48 ppm) and lactate (1.33 ppm) levels were higher in the low PET type (Fig. 4A). We performed receiver operating characteristic (ROC) analysis to determine how well these 4 metabolites predict the performance of PET criteria diagnosis (Fig. 4B). Glucose had the highest area under the curve (AUC) value at 0.968 (sensitivity = 0.897; specificity = 0.955). Creatinine had an AUC value of 0.776 (sensitivity = 0.655; specificity = 0.909), lactate had an AUC value of 0.712 (sensitivity = 0.862; specificity = 0.5), and alanine had an AUC value of 0.679 (sensitivity = 0.483; specificity = 0.818) in single ROC analysis. The AUC value of the combination of the 4 markers (0.975) with multiple ROC analyses was higher than that of each of the metabolites, implying that these 4 metabolites account for most of the metabolic difference between the high and low PET types. The plot of the OPLS-DA model score revealed non-significant differences between the high average and low average PET types (R^2^ = 0.344, Q^2^ = 0.155; Supplemental Table 2). However, there were concentration trends among the four groups: when the PET type was low, glucose and lactate increased and creatinine and alanine decreased (Supplemental Fig. 2). We additionally performed calibration of each marker by the dwell time in the 122 patients who had data on overnight PD effluent dwell time. As a result, a similar tendency was confirmed when compared to the entire data without dwell time correction (Supplemental Fig. 3).

S-TOCSY and OPLS-DA analyses were also performed in groups excluding icodextrin users (Supplemental Table 3). The results were similar to those of the overall 125 patients. The plot of the OPLS-DA model score revealed statistically significant differences between high and low PET types (R2 = 0.755, Q2 = 0.669; Supplemental Fig. 4B). The relative concentrations of alanine and creatinine in S-TOCSY were higher in the high PET type than in the low PET type. Furthermore, the low PET type had higher relative concentrations of glucose and lactate than the high PET type (Supplemental Fig. 4A). In addition, the individual marker analysis was not significantly different compared to those of the overall 125 patients (Supplemental Fig. 5).

### Correlation between measured PET values and the total NMR signal

**Figure 5:**
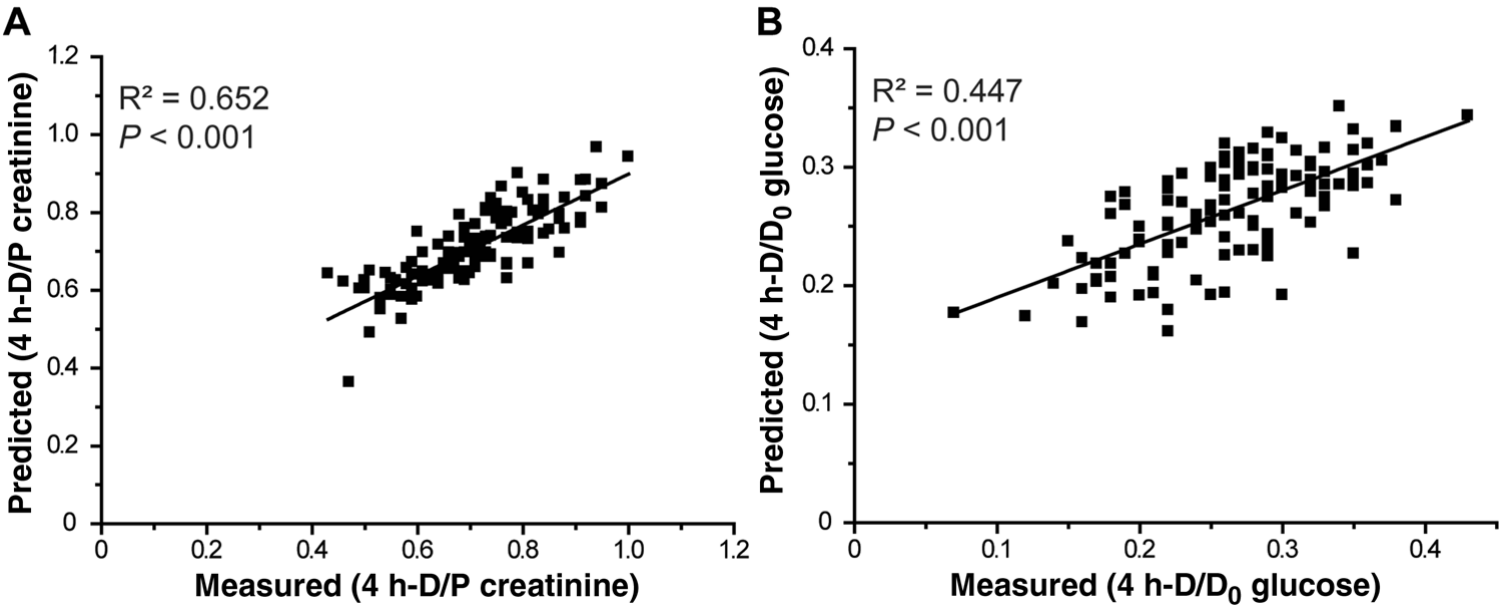
Prediction of measured PET results from total NMR signals. Prediction model between total NMR signals and measured PET results: (**A**) 4 h-D/P creatinine and (**B**) 4 h-D/D_0_ glucose. The Y-axis corresponds to the predicted values that were calculated using the PLS regression followed by total NMR signal prediction. The X-axis corresponds to the measured 4 h-D/P creatinine (**A**) and 4 h-D/D_0_ glucose (**B**) for determining the PET. The black line represents the best fit between the predicted and observed PET results. *PET* peritoneal equilibration test, *PLS* partial least square, *NMR* nuclear magnetic resonance.

We tested whether the PET results, the 4 h-D/P creatinine or 4-h to 0-h dialysate glucose ratio (4 h-D/D_0_ glucose) measured in the hospital, is correlated with the total NMR metabolomics profile of overnight PD effluents. Partial least square (PLS) regression analysis showed a significant correlation between the measured PET results (dependent variable) and total NMR metabolomics profile (independent variable) [dialysate-to-plasma creatinine ratio at the 4-h dwell time (4 h-D/P creatinine): R^2^ = 0.652, Q^2^ = 0.538; 4 h-D/D_0_ glucose: R^2^ = 0.447, Q^2^ = 0.381; Fig. 5A,B]. These data suggest that, in addition to the individual markers above, total NMR metabolomic profile might be useful in predicting the PET type of patients.

### Metabolomic profiles according to the presence of DM

Since there were more diabetic patients in the high PET types, we performed a subgroup analysis according to the presence or absence of DM. Multivariate analysis showed significant metabolic differences between high and low PET types in the DM (R^2^ = 0.739, Q^2^ = 0.454) and non-DM groups (R^2^ = 0.759, Q^2^ = 0.613) according to the OPLS-DA score plot (Supplemental Fig. 6A,B). In addition, measured PET results correlated well with total NMR signals in patients with DM (4 h-D/P creatinine: R^2^ = 0.599, Q^2^ = 0.432; 4 h-D/D_0_ glucose: R^2^ = 0.559, Q^2^ = 0.373;7A,B) and those without DM (4 h-D/P creatinine: R^2^ = 0.549, Q^2^ = 0.433; 4 h-D/D_0_ glucose: R^2^ = 0.463, Q^2^ = 0.369; Supplemental Fig. 7C,D). These data suggest that the NMR metabolomics could be applied even in the presence or absence of diabetes to predict the PET results.

## Discussion

In the present study, NMR based-metabolomic profiles using OPLS-DA showed a significant difference between high and low PET types for overnight PD effluents. Creatinine and alanine were contributing metabolites in the high PET type, whereas glucose and lactate were contributing metabolites in the low PET type. Notable correlation was observed between measured 4 h-D/P creatinine and 4 h-D/D_0_ glucose PET results with total NMR signals.

Metabolomics techniques systematically detect multiple metabolites directly from complex biological samples^27^. Measurement of a wide range of metabolites could provide a more accurate assessment of the complex clinical phenotype of patients than allowed by the conventional analytes. The PD effluent contains exogenous and endogenous metabolites, proteins, free-floating cells, and nucleic acids, which are all available for sampling as a non-invasive liquid biopsy without burden to the patients with PD^28^. Although PD effluents are readily available biofluids, there have not been many metabolomic studies for PD. A pilot study applied untargeted metabolomics to identify metabolites in PD effluents^20^. Several studies were conducted to evaluate peritoneal membrane dysfunction in patients with PD by applying metabolomics technologies^29^. In the targeted metabolomic study, Wiesenhofer et al. investigated PD effluent profiles of patients with PD and evaluated the cytoprotective effect of alanyl-glutamine supplementation to PD fluid^28^. Previously, there were few studies on the peritoneal membrane transport characteristics and PET type of patients with PD by metabolomics analysis of PD effluents, especially overnight PD effluents.

The PET is a useful examination of peritoneal transport characteristics and is used for PD prescription. It is recommended to be conducted at a minimum of 4 weeks after initiation of PD because earlier testing may not accurately reflect the peritoneal transport status of patients established on PD^2^. Additionally, peritoneal membrane function should be monitored regularly, once a year, and additional tests should be conducted in clinically indicative situations, including uncontrolled volume overload, decreased net ultrafiltration volume, acceleration of HTN, and change of solute removal, etc^2,30^. Evaluation of peritoneal membrane function is also important because it is an independent predictor of patient survival^6^. In these regards, it is important to perform the PET regularly, but it takes a long time to perform and the required multiple sampling is inconvenient. The PET is a method to confirm the characteristics of the peritoneal membrane, and it measures only the change in the diffusion rate of solute that occurs after various changes in the peritoneum with glucose and creatinine. Therefore, it would be meaningful to be able to predict peritoneal membrane characteristics with overnight PD effluents using metabolomic analysis. Currently, NMR metabolomic data acquisition costs ~ 20 USD per sample, and the downstream analysis can be easily automated with software, particularly with the suggested markers, i.e., glucose, alanine, creatinine, and lactate. If our results are validated in larger cohorts, there may be a possibility that NMR measurement of PD effluents may be a convenient option for PD patients. An example of NMR profiling used in clinical settings would be the NMR LipoProfile^®^ test that measures individual lipoprotein species more conveniently than traditional ultracentrifugation methods^31–33^.

Herein, the contributing metabolites for the high PET type were creatinine and alanine, whereas the contributing metabolites for the low PET type were glucose and lactate. The results were similar when normalized by the PD effluent dwell time. In addition, metabolomic profiles were similar when the analysis was performed in the group excluding icodextrin users. In the high PET type, the solute transport is fast, so it can be seen that the overnight dialysis effluents contain lots of creatinine, a representative small uremic toxin compared to that in the low PET type. In the low PET type, less glucose and lactate of the dialysate was absorbed due to slow solute transport, leaving more glucose and lactate in the dialysis effluents than in the high PET type. Alanine was higher in the PD effluent of patients with the high PET type than in those with the low PET type. Alanine is the second most abundant amino acid in circulation, involved in protein production, and used as an energy source in muscles and the central nervous system^34^. It is also involved in the immune system and plays a role in T cell activation^34^. In a previous study, serum metabolites were different between patients with PD and hemodialysis^14^. The PD group was associated with higher levels of alanine, which is linked to glucose metabolism, than the control and hemodialysis groups^14^. In another study, when PD effluent was analyzed using ^1^H-^13^C NMR spectroscopy, it was found to be rich in amino acids, including alanine^35^. Dunn et al. showed metabolomic changes in PD effluent in patients who develop encapsulating peritoneal sclerosis^19^. Changes in amino acids, amines and derivatives, short-chain fatty acid and derivatives, and sugars were observed prior to the development of encapsulating peritoneal sclerosis. Six amino acids, including beta-alanine/alanine, were detected at higher relative concentrations in the PD effluent from patients with encapsulating peritoneal sclerosis. As such, the detection of alanine in PD effluent may be a change during PD, and increased amino acids can be observed during cell damage through proteolysis or release of free amino acids. In this regard, rat peritoneal mesothelial cells have been shown to produce amino acids, such as alanine, glutamine, glycine, threonine, and serine, when cultured with the hypertonic medium^36^. The detection of more alanine in the high transporter compared to the low transporter in our study suggests changes and damage to the peritoneal membrane in patients with the high transporter type.

In the present study, we predicted modified PET types using overnight PD effluents in a rapid, non-invasive manner with NMR metabolomics. NMR is one of the most widely used analytical techniques in metabolomics research, with various benefits including excellent repeatability, sample recovery, and non-destructive sample handling. The overnight PD effluents are easier to obtain than samples during PET. In addition, since PD directly contacts the action site, which is a peritoneal membrane, it can reflect the peritoneal characteristics of patients with PD. However, there are a couple of limitations to this study. First, there were not distinguishable metabolites between the average PET types (high average or low average) and the high and low PET types in the present study. However, clinically, it can be a problem for patients with a high or low PET type compared to patients with an average PET type to maintain PD. This is because patients with a high PET type have problems with increasing ultrafiltration failure, mortality, and technique failure, and those with a low PET type have other problems, such as underdialysis. Therefore, even if it is impossible to distinguish all PET types, it is clinically meaningful to distinguish between patients with a high or low PET type. Second, this study was conducted in only Koreans, so further studies on other races and more patients are needed.

In conclusion, creatinine and alanine were contributing metabolites in the high PET type, whereas glucose and lactate were contributing metabolites in the low PET type. Measured PET results correlated well with total NMR signals.

## Materials and methods

### Study design and population

The present study was a single-center retrospective analysis of prospectively collected registry data. Two hundred twenty-three patients older than 18 years of age started PD at Seoul National University Hospital from January 2012 to February 2016 (Fig. 1). Among them, 93 patients were excluded due to unavailable overnight PD effluents. Overnight PD effluents were harvested from 130 patients on the day of the first PET after PD initiation. In total, 125 patients were analyzed, after excluding 5 patients who underwent a standard PET (2.5% PET), instead of a 4.25% dextrose modified PET. This study was approved by the Seoul National University Hospital Institutional Review Boards (No. 1506-097-681). All study patients provided written informed consent. All clinical investigations were conducted in accordance with the guidelines of the 2008 Declaration of Helsinki.

### Clinical data collection

The baseline demographic and PD-related data, such as age, sex, comorbidities, etiology of end-stage kidney disease, and biochemical data, immediately before PD catheter insertion were collected. PET and PD adequacy data performed 1 month after PD initiation were also collected. Blood levels of hemoglobin, albumin, and C-reactive protein were measured using routine laboratory methods. Creatinine was measured using the isotope dilution mass spectrometry (IDMS) reference method^37^. PD effluent concentrations of creatinine and glucose were measured by validated standard methods in the clinical laboratory of the Seoul National University Hospital. Creatinine concentrations of PD effluent were corrected for high glucose levels by determining a correction factor from measurements of serial dilution of unused PD fluid.

### PET

The PET was usually performed 4 weeks after PD initiation at our center, and a modified 4.25% dextrose PET was conducted^9^. A 1.5% dextrose dialysis solution (1.5–2 L) was instilled on the night before the PET and was drained immediately before the PET test. This overnight effluent was harvested for future analysis. The peritoneal transporter type was categorized according to the 4 h-D/P creatinine during the PET as follows: high (> 0.82), high average (0.72–0.82), low average (0.62–0.72), or low transporter (< 0.62)^38^.

### Sampling and sample preparation for metabolomics

Overnight PD effluents on the night before the first PET were collected and frozen at − 80 °C, within 24 h. Before the NMR experiment, frozen PD effluent samples were thawed and transferred to 1.5-mL Eppendorf tubes and centrifuged at 17,000 rpm for 15 min at 4 °C. After centrifuging, the supernatant of 450 μL was mixed with 50 μL of D_2_O containing a final concentration of 0.025% sodium-3-trimethylsilylpropionic acid (TSP; Cambridge Isotope Lab) as an internal standard, and 5-mM NaH_2_PO_4_ and 2-mM Na_2_HPO_4_ as a pH buffer.

### Data processing and analysis of the NMR spectra and multivariate pattern recognition

All NMR spectra were acquired using the 850-MHz NMR spectrometer (Bruker AVNACE III HD) equipped with a cryogenic probe (National Center for Inter-University Research Facilities, Seoul National University). NMR acquisition occurred at 27 °C using the “noesypr1d” sequence with the following parameters: spectral width (SW): 15.9804 ppm along the 1H dimensions; O1P = 4.697 ppm; number of scan (NS) = 64; relaxation delay time = 2 s; and mixing time = 0.01 s.

Phase correction and baseline subtraction were performed with the M-Nova program (Mestrelab Research). The peak ppm values were standardized with reference to the TSP signal at 0 ppm. The processed NMR spectra were exported to ASCII text files and binned with 0.037-ppm intervals. The water-related regions (4.71–5.1 ppm) were excluded from all spectra. Using an in-house Perl program, binning, normalization, and conversion were conducted.

Chenomx (Edmonton, Alberta, Canada) and an inhouse-built NMR spectrum database were used to perform spectral identification and quantification. Multivariate analyses were conducted with Origin (OriginLab MA) and SIMCA-P 11.0 (Umetrics, Sweden). Markers were analyzed using ROC analysis using Prism (version 9.0.0, GraphPad Software). The markers were statistically analyzed by the Benjamini–Hochberg correction. Pearson correlation analysis was used to determine the relationship between total NMR signals and the grading of PET types on routine testing. The quality of the models was shown based on R^2^ and Q^2^ values. R^2^ is defined as the proportion of variance in the data explained by the models and presents goodness of fit, and Q^2^ is defined as the proportion of variance in the data predicted by the model and presents predictability.

### Statistical analysis

Demographic continuous variables are presented as mean ± standard deviation and were compared between PET types using one-way analysis of variance. Nonparametric variables are expressed as median (interquartile range) and were compared using the Kruskal–Wallis test. Categorical variables were analyzed using the chi-square or Fisher’s exact test, and the results are presented as frequency and percentage. Differences in biochemical parameters among PET types were analyzed using SPSS statistical software (SPSS version 20.0, IBM Co., Armonk, NY, USA). *P-*values < 0.05 were considered statistically significant.

## Supplementary Information

Supplementary Information.

## Data Availability

All data generated or analyzed during this study are included in this published article, supplementary data.
