# Supplementary material for: Metabolomic profiling of overnight peritoneal dialysis effluents predicts the peritoneal equilibration test type
Source: Sci Rep. 2023 Mar 7;13:3803. doi: 10.1038/s41598-023-29741-3 (PMC9992441; doi:10.1038/s41598-023-29741-3)
Supplement: Supplementary file 1 — Supplementary Information. [file 41598_2023_29741_MOESM1_ESM.docx]

**Supplemental Material**

**Supplemental Table 1. Metabolites identified from the PD effluents**

| Metabolite | ppm (parts per million) |
| --- | --- |
| 3-Hydroxybutyrate | 1.15 (d), 2.31 (m), 2.41 (m), 4.16 (m) |
| Acetate | 1.91 (s) |
| Alanine | 1.47 (d), 3.79 (q) |
| Creatinine | 3.11 (s), 4.06 (s) |
| Glucose | 3.22 (m), 3.38–3.91 (m), 5.23 (d) |
| Glutamine | 2.09 (m), 2.44 (m), 3.70 (t) |
| Isoleucine | 0.94 (t), 1.02 (d) |
| Lactate | 1.33 (d), 4.09 (q) |
| Leucine | 0.97 (t), 1.72 (m) |
| Lysine | 1.72 (m), 1.92 (m), 3.02 (t) |
| Maltose | 3.26 (m), 3.42 (m), 3.62–3.91 (m), 4.64 (d), 5.4 (d, d) |
| Proline | 2.02 (m), 2.35 (m), 4.12 (m) |
| Threonine | 1.32 (d), 4.25 (m) |
| Valine | 0.99 (d), 1.04 (d), 2.27 (m) |

The scale of ppm in NMR indicates the relative positions of resonances in the NMR spectrum.

S, singlet; d, doublet; t, triplet; q, quartet; m, multiplet; PD, peritoneal dialysis; NMR, nuclear magnetic resonance.

**Supplemental Table 2. Q^2^ results of OPLS-DA analysis according to PET types in 125 patients overall**

| Group | Q^2^ value of OPLS-DA |
| --- | --- |
| High vs. Low | 0.666 |
| High vs. Average | 0.129 |
| High vs. High average | 0.073 |
| High vs. Average & low | 0.163 |
| Low vs. Average | 0.268 |
| Low vs. Low average | 0.150 |
| Low vs. Average & high | 0.281 |
| High average vs. Low average | 0.155 |

PET types were categorized according to the criteria described in the methods, and the table shows the Q2 results of OPLS-DA analysis for each matching type. Also, the average group includes both high and low averages.

OPLS-DA, orthogonal projections to latent structure-discriminant analysis; PET, peritoneal equilibration test

**Supplemental Table 3. Q^2^ results of OPLS-DA analysis according to PET types in the group excluding icodextrin users**

| Comparison among PET types | Q^2^ value of OPLS-DA |
| --- | --- |
| High vs. Low | 0.669 |
| High vs. Average | 0.0713 |
| High vs. High average | N/A |
| High vs. Average & low | 0.0314 |
| Low vs. Average | 0.22 |
| Low vs. Low average | 0.143 |
| Low vs. Average & high | 0.231 |
| High Average vs. Low average | N/A |

PET types were categorized according to the criteria described in the methods, and the table shows the Q2 results of OPLS-DA analysis for each matching type. Also, the average group includes both high and low averages.

OPLS-DA, orthogonal projection to latent structure-discriminant analysis; PET, peritoneal equilibration test; N/A, not applicable

**Supplemental Figure 1. PCA plot derived from NMR spectra of overnight PD effluents** **among PET types**

**
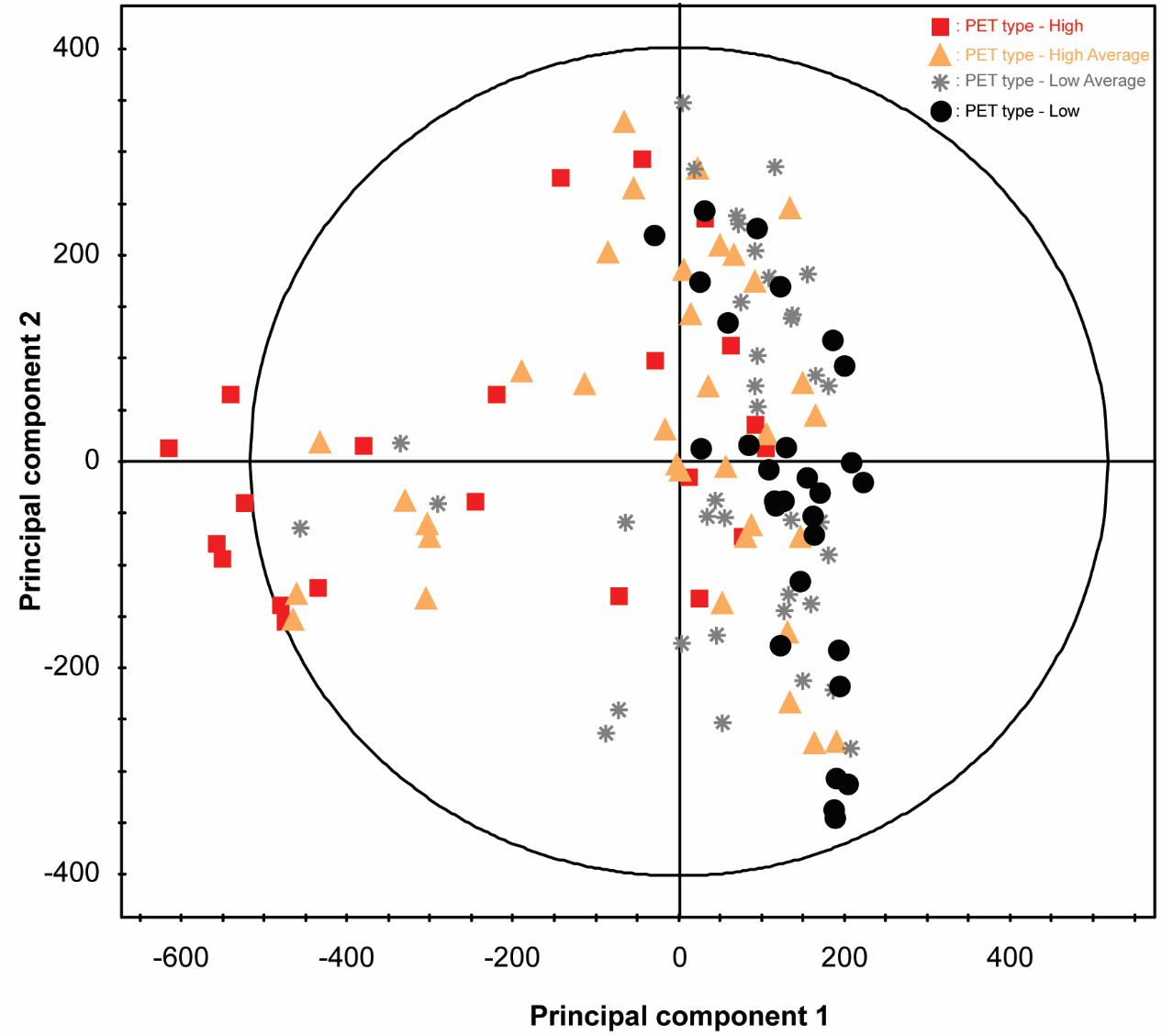
**

PCA plot showing the principal component 1 (X-axis) and principal component 2 (Y-axis) of the model. Each point represents a metabolite profile of each sample (R^2^ = 0.926, Q^2^ = 0.809).

PCA, principal component analysis; NMR, nuclear magnetic resonance; PET, peritoneal equilibration test; PD, peritoneal dialysis.

**Supplemental Figure 2.** **Statistically significant differences in markers among the PET types**

**
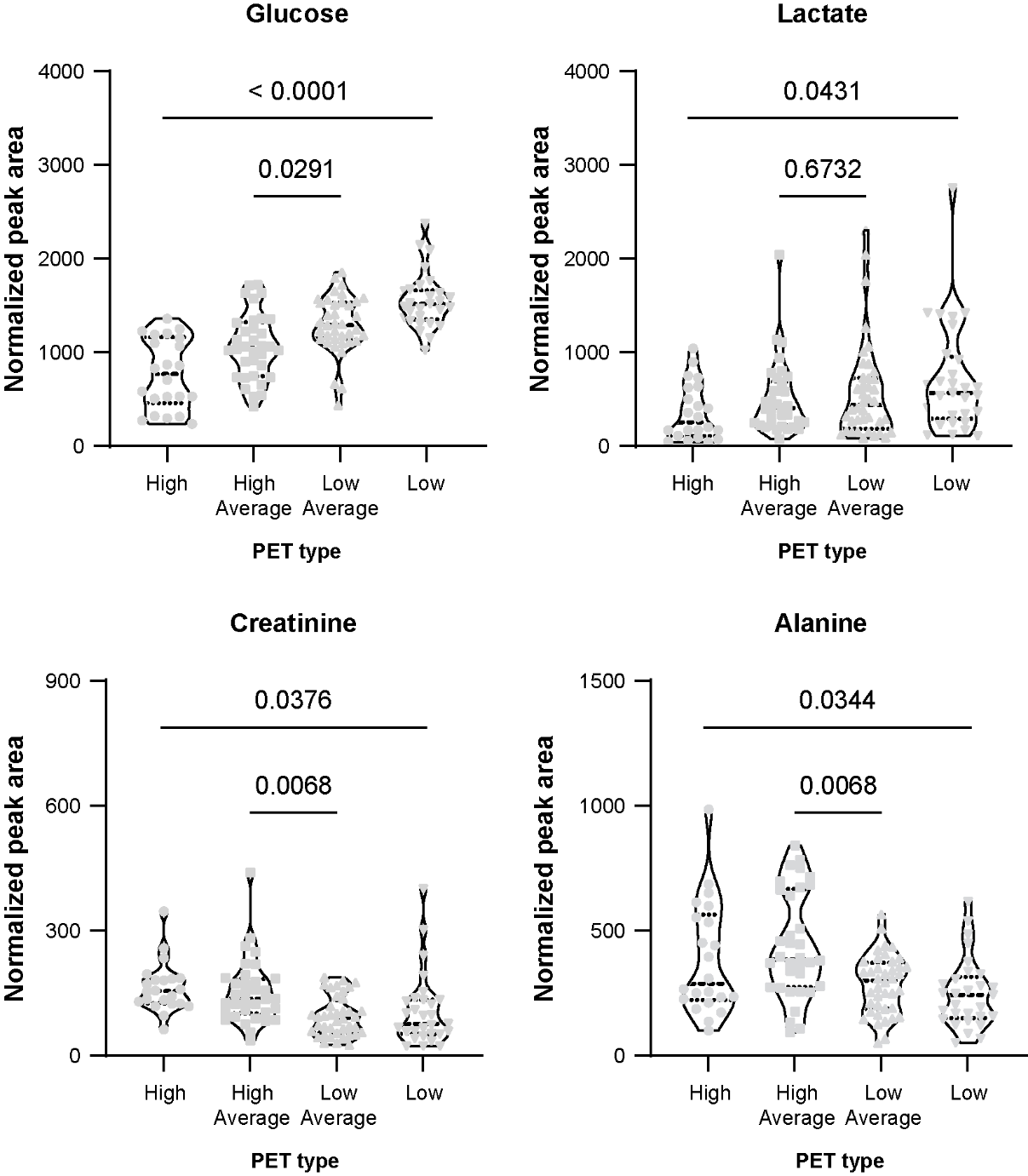
**

Levels of the markers among the PET types.

Violin plots are presented with the median and quartiles. The levels were determined as the mean peak areas normalized by the total area based on the 1D proton NMR spectra in Figure 2A.

PET, peritoneal equilibration test; NMR, nuclear magnetic resonance; PLS-DA, partial least square-discriminant analysis; ROC, receiver operating characteristic; AUC, area under the curve

**Supplemental Figure 3.** **Statistically significant differences in markers among the PET types normalized by dwell time**

**
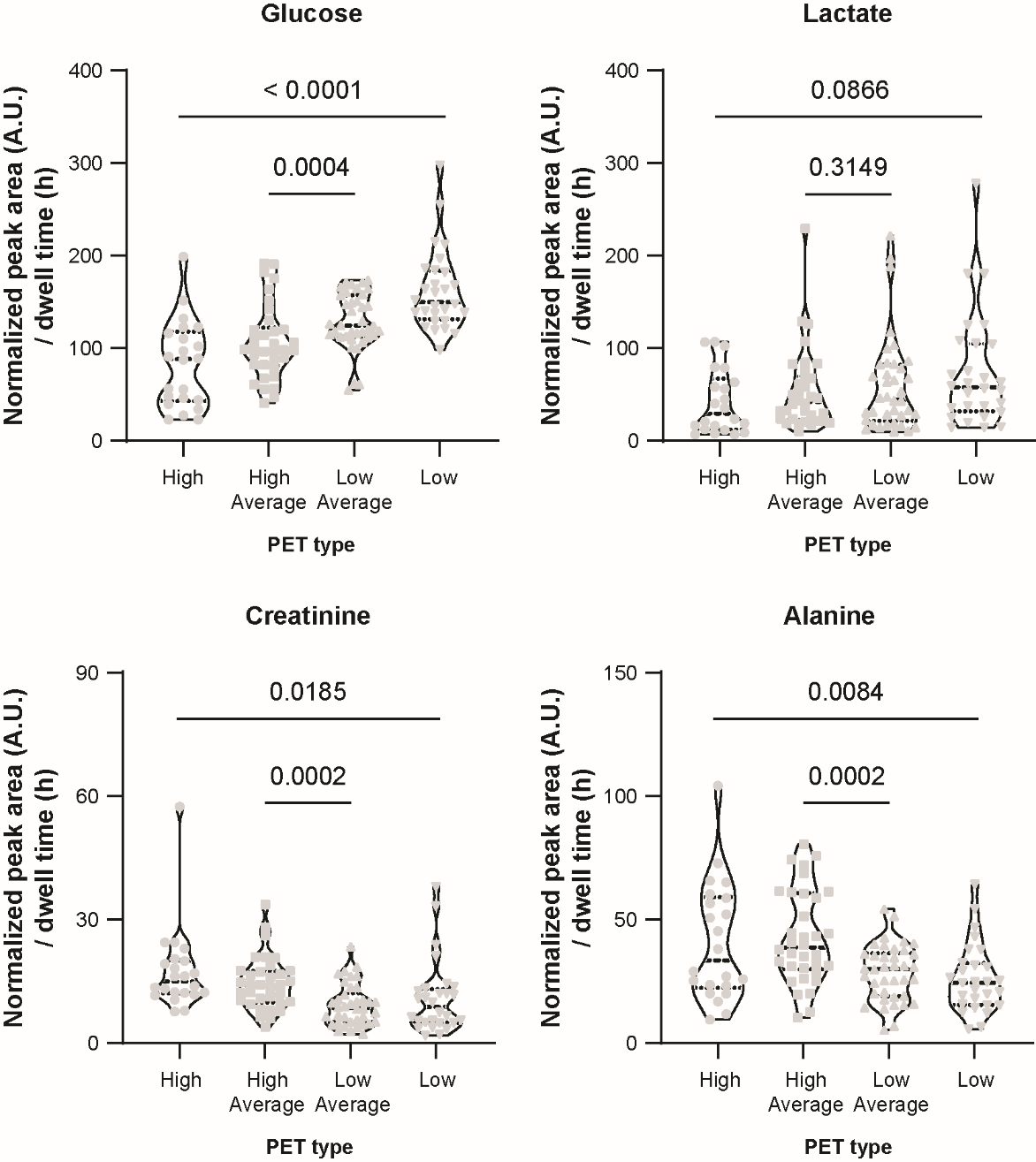
**

Levels of the markers among the PET types.

Violin plots are presented with the median and quartiles. The levels were determined as the mean peak areas normalized by the total area based on the 1D proton NMR spectra in Figure 2A.

PET, peritoneal equilibration test; NMR, nuclear magnetic resonance; PLS-DA, partial least square-discriminant analysis; ROC, receiver operating characteristic; AUC, area under the curve.

**Supplemental Figure 4.** **S-TOCSY and** **OPLS-DA score plots derived from the NMR spectra based on the separation between the high and low PET types in the group that excluded icodextrin users**


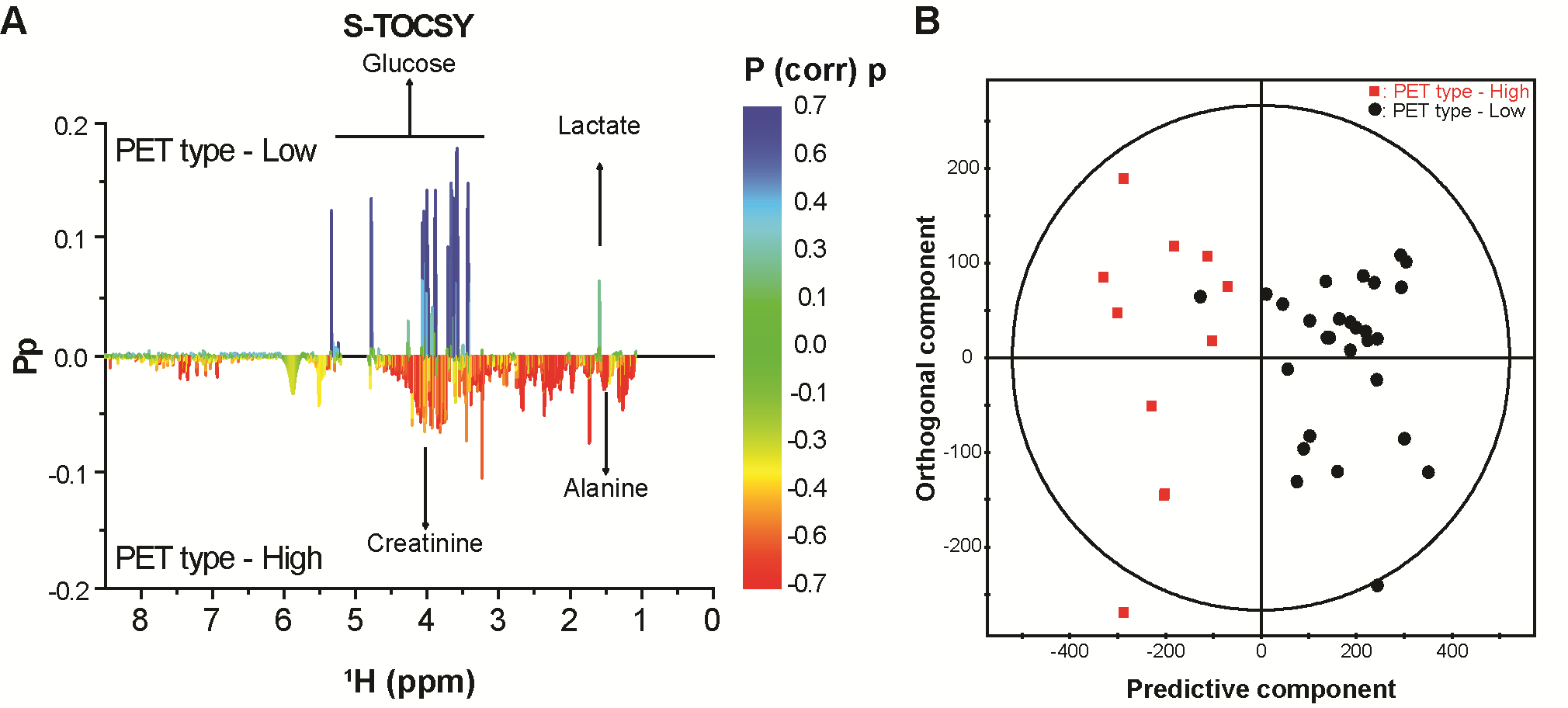


S-TOCSY from the OPLS loading plot indicates the model coefficients for the NMR variables. (A). The OPLS-DA score plot derived from the NMR spectra of overnight PD effluents among patients excluding icodextrin users (B). The OPLS-DA score plot showing the predictive component (X-axis) and orthogonal component (Y-axis) of the model. Each symbol represents the metabolic characteristic of a particular sample in the projected dimensions of the predictive and orthogonal components. The separation along the predictive component axis indicates the difference of interest (high vs. low PET type) from the metabolic profile.

S-TOCSY, statistical total correlation spectroscopy; OPLS-DA, orthogonal projection to latent structure discriminant analysis; NMR, nuclear magnetic resonance; PET, peritoneal equilibration test

**Supplemental Figure 5.** **Significant differences in markers among the group excluding icodextrin users**


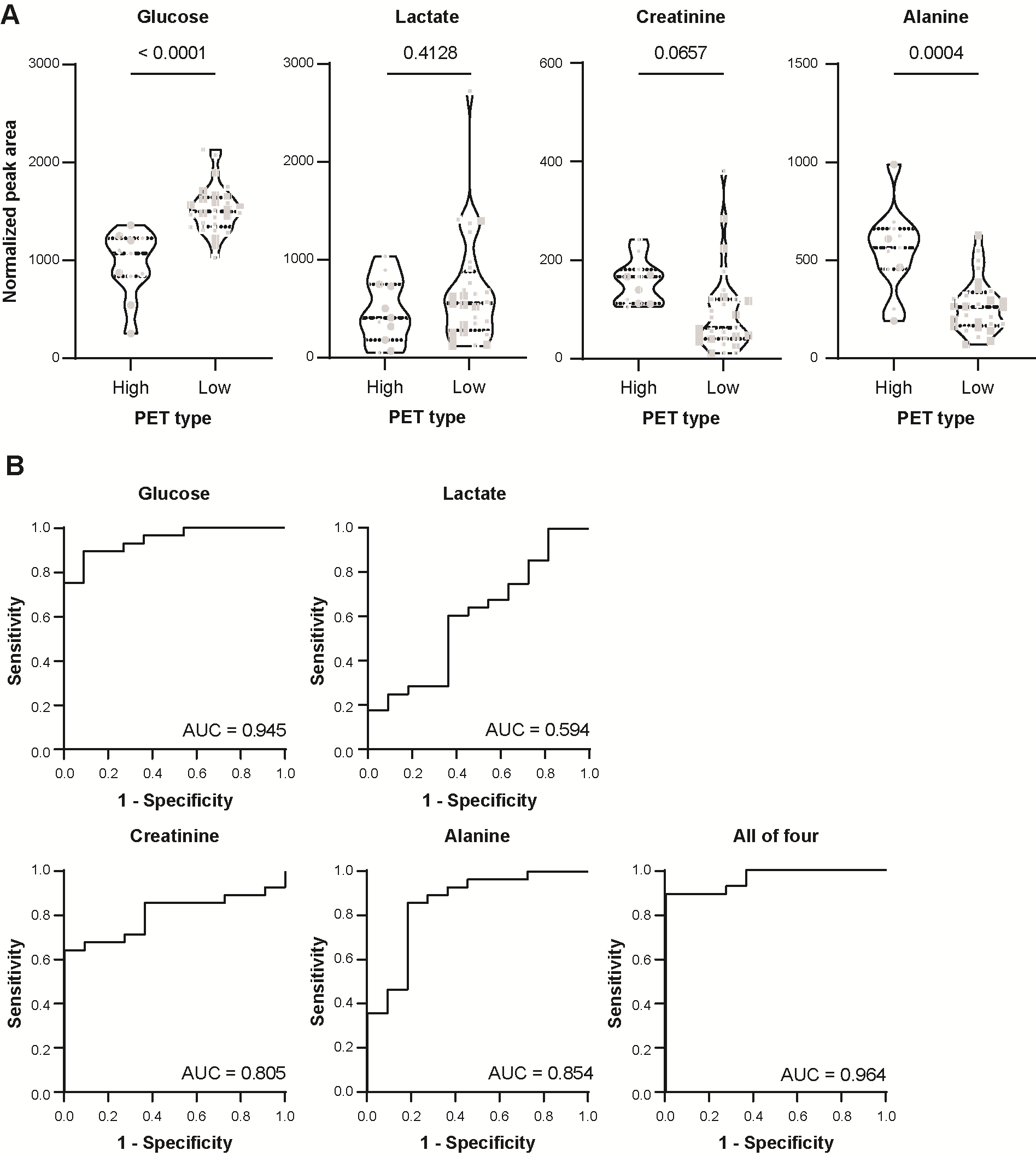


(A) Levels of markers between the high and low PET types.

Violin plots are presented with the median and quartiles. The levels were determined as the mean peak areas normalized by the total area based on the 1D proton NMR spectra in Supplemental Figure 2A.

(B) PLS-DA-based ROC curves of the diagnosis of the high and low PET types for the individual markers–glucose, lactate, creatinine, and alanine, as well as the combination of the four markers.

Statistical analysis of the markers was performed using the Benjamini–Hochberg correction, and the resulting FDR *P*-values are indicated.

PET, peritoneal equilibration test; NMR, nuclear magnetic resonance; PLS-DA, partial least square-discriminant analysis; ROC, receiver operating characteristic; AUC, area under the curve; FDR, false discovery rate

**Supplemental Figure 6.** **OPLS-DA score plot derived from NMR spectra between the high and low PET types according to the presence of DM**


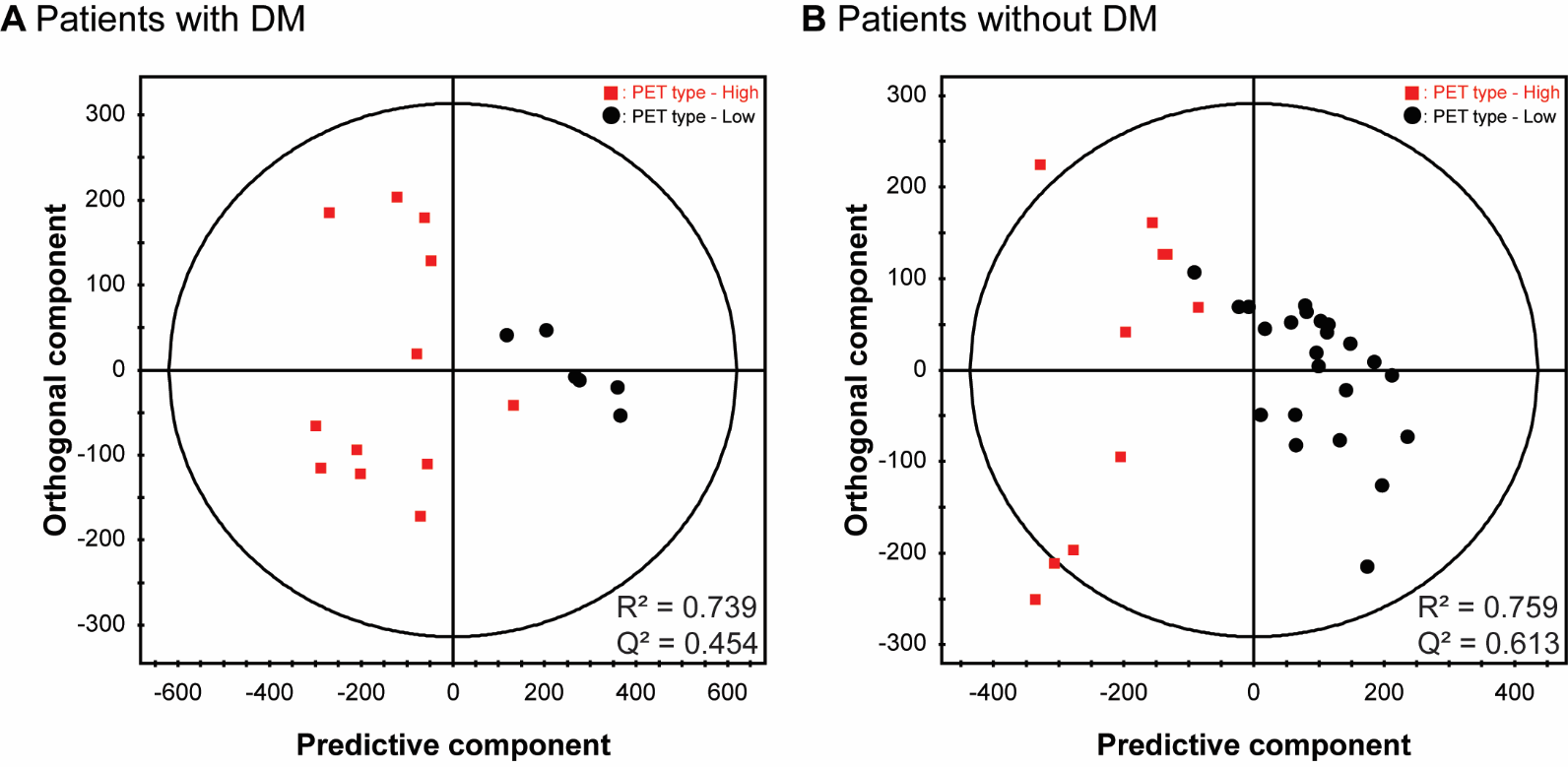


OPLS-DA score plot derived from NMR spectra of overnight PD effluents in patients with DM (A) and those without DM (B). OPLS-DA score plot showing the predictive component (X-axis) and orthogonal component (Y-axis) of the model. Each symbol represents the metabolic characteristic of a particular sample in the projected dimensions of the predictive component and orthogonal component. The separation along the predictive component axis indicates the difference of interest (high vs. low PET type) from the metabolic profile.

OPLS-DA, orthogonal projections to latent structure discriminant analysis; NMR, nuclear magnetic resonance; PET, peritoneal equilibration test; DM, diabetes mellitus.

**Supplemental Figure 7.** **Prediction of measured PET results from total NMR signals according to the presence of DM**

**
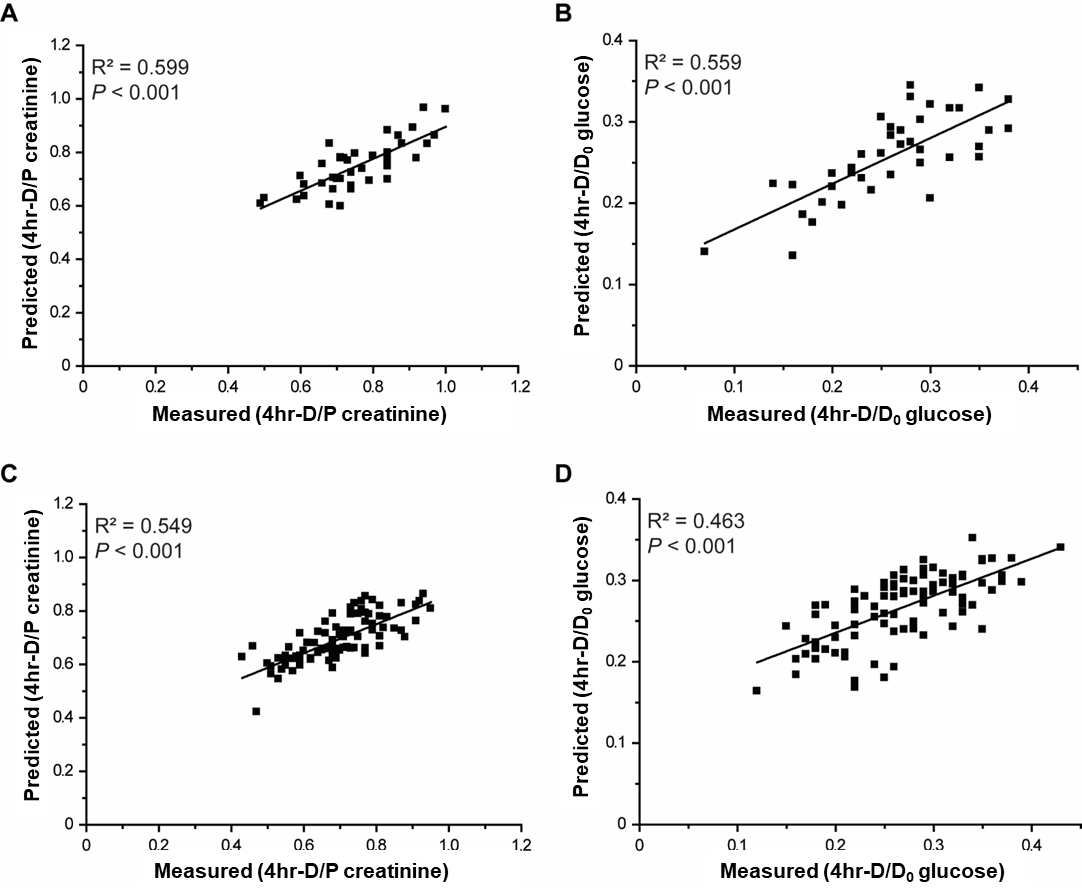
**

(A–B) Prediction model between total NMR signals and measured PET results in patients with DM.

Prediction model between total NMR signals and PET criterion: (A) 4hr-D/P creatinine and (B) 4hr-D/D_0_ glucose. The Y-axis corresponds to the predicted values that were calculated by using PLS regression followed by total NMR signal prediction. The X-axis corresponds to the measured 4hr-D/P creatinine (A) and 4hr-D/D_0_ glucose (B) for determining the PET. The black line represents the best fit between the predicted and observed PET results.

(C–D) Prediction model between total NMR signal and PET criterion without DM patients.

Prediction model between total NMR signals and measured PET results: (C) 4hr-D/P creatinine and (D) 4hr-D/D_0_ glucose. The Y-axis corresponds to the predicted values that were calculated using PLS regression followed by total NMR signal prediction. The X-axis corresponds to the measured 4hr-D/P creatinine (C) and 4hr-D/D_0_ glucose (D) for determining the PET. The black line represents the best fit between the predicted and observed PET results.

PET, peritoneal equilibration test; PLS, partial least square; NMR, nuclear magnetic resonance; DM, diabetes mellitus.
